# Can We Rely on Projections of the Immigrant Population? The Case of Norway

**DOI:** 10.1007/s10680-023-09675-2

**Published:** 2023-11-13

**Authors:** Nico Keilman

**Affiliations:** Department of Economics, University of Oslo, Oslo, Norway

**Keywords:** Stochastic forecast, Immigrants, Second generation, Random share method, Prediction interval

## Abstract

Demographic forecasters must be realistic about how well they can predict future populations, and it is important that they include estimates of uncertainty in their forecasts. Here we focus on the future development of the immigrant population of Norway and their Norwegian-born children (“second generation”), grouped by three categories of country background: 1. West European countries plus the United States, Canada, Australia, and New Zealand; 2. Central and East European countries that are members of the European Union; 3. other countries. We show how to use a probabilistic forecast to assess the reliability of projections of the immigrant population and their children. We employ the method of random shares using data for immigrants and their children for 2000–2021. We model their age- and sex-specific shares relative to the whole population. Relational models are used for the age patterns in these shares, and time series models to extrapolate the parameters of the age patterns. We compute a probabilistic forecast for six population sub-groups with immigration background, and one for non-immigrants. The probabilistic forecast is calibrated against Statistics Norway’s official population projection. We find that a few population trends are quite certain: strong increases to 2060 in the size of the immigrant population (more specifically those who belong to country group 3) and of Norwegian-born children of immigrants. However, prediction intervals around the forecasts of immigrants and their children by one-year age groups are so wide that these forecasts are not reliable.

**Supplementary Information:**

The online version contains supplementary material available at 10.1007/s10680-023-09675-2.

## Introduction

Forecasts of the immigrant population are essential for government planning with respect to labour market and health policy, integration issues, and educational facilities. The future of this population sub-group is uncertain, but some developments are more likely than others. Therefore, probabilistic forecasts are a necessary tool for informed planning and decision making by policy makers.

Statistics Norway publishes projections for the population divided by age, sex, and migration back-ground at regular intervals. The most recent projections were published in July 2022; see Thomas and Tømmerås (2022). These projections are deterministic; uncertainty is accounted for by formulating several scenarios for the future development of fertility, mortality, and international migration. Whereas a scenario approach may be useful in case one is interested in future population trends based on a set of specific assumptions, the deterministic nature of the scenarios implies that uncertainty is not quantified. This makes it difficult for the user to choose between the different scenarios. Also, when the user just selects the scenario results labelled as most likely by the producer of the projections, this may be a choice that is far from optimal. Take the example of a planner of educational facilities: under-predicting the number of schoolchildren may lead to hiring extra capacity, which may cost more than idle capacity in case of over-predictions. In such cases, the optimal choice is a trajectory a little or very much higher than the most likely trajectory—how much higher depends on the expected variation in the predictions. All this suggests a probabilistic forecast, not a deterministic one. Indeed, the Norwegian Ministry of Finance (more precisely, its Advisory Committee on Models and Methods), which is responsible for designing the country’s long-term economic plans, has proposed that Statistics Norway compute a probabilistic population forecast. One should note that the aim of a probabilistic forecast is not to present estimates of future trends that are more accurate than those computed in a deterministic forecast, but rather to give the user a better picture of prediction uncertainty.

The literature on stochastic demographic forecasts includes multi-country forecasts (see United Nations (2022) for all countries of the world, and Alho et al. (2006) for 18 European countries), forecasts for national populations (for early contributions see Lee & Tuljapurkar, 1994; Alho, 1998; Keilman et al., 2002), for multiregional and subnational populations (Wilson, 2013a, 2013b; Wiśniowski & Raymer, 2016; Yu et al., 2023), for households (Alders, 1999, 2001; Alho & Keilman, 2010; Christiansen & Keilman, 2013; De Beer & Alders, 1999, 1999; Keilman, 2016; Scherbov & Ediev, 2007), for the labour market (Fuchs et al., 2018), and for long-term care (Vanella et al., 2020). As to immigrant populations, statistical agencies and individual authors have computed *deterministic* forecasts for this population sub-group (see Rees, 2009 for a review), but very few have quantified the uncertainty surrounding future developments of immigrants. For exceptions, see Alders (2005), Coleman and Scherbov (2005), and a forecast published by Statistics New Zealand, to be discussed later. The aim of the current paper is to contribute to the literature, and to compute a probabilistic forecast for the migrant population of Norway.

We use a method that combines a probabilistic cohort-component forecast with random shares. The shares distribute each age-sex specific forecast result over various sub-populations. The method has been employed for computing stochastic household forecasts. The first applications (Alders, 1999, 2001; De Beer & Alders, 1999; Scherbov & Ediev, 2007; Wilson, 2013a, 2013b) based uncertainty parameters on subjective reasoning. In later studies (Alho & Keilman, 2010; Christiansen & Keilman, 2013; Keilman, 2016), uncertainty parameters were estimated from data.

Few probabilistic forecasts of immigrant populations exist. One has been reported by Alders (2005), but the author presented results only, not the method. The approach of Coleman and Scherbov (2005) relied heavily on expert opinions. The authors started with a deterministic cohort-component projection of the population of the UK from 2001 to 2100. The population was broken down into four ethnic groups: White, Asian, Black, and Mixed. High, Medium, and Low scenarios were formulated for future values of the total fertility rate, life expectancy at birth, and net migration. Subjectively chosen probabilities were assigned to the High-Low intervals for each of these three random variables in the years 2001, 2021, 2051, and 2100, while the Medium scenario was chosen as the mean of the distribution. The values at intermediate dates were determined using linear interpolation, and the results of 1 000 random simulations were analysed. Statistics New Zealand published stochastic ethnic population forecasts by age and sex for the years 2018–2043 and eight ethnic groups; see https://nzdotstat.stats.govt.nz/WBOS/Index.aspx?DataSetCode=TABLECODE8613. Some technical details are given at https://datainfoplus.stats.govt.nz/item/nz.govt.stats/0790e9b3-4cfe-4ac5-a23a-6e8864ff8d5c/3. For each ethnic group, total fertility and life expectancy are modelled as a Random Walk with Drift, whereas ARIMA-type of time series model are used for migration.

We compute a probabilistic forecast for the immigrant population of Norway and their Norwegian-born children (“second generation”) broken down by age and sex. We adapt the random share method discussed earlier to data for the population with immigrant background. We distinguish both the immigrants and their children according to three groups of countries, see Sect. 2. The population without any migration background forms a seventh population subgroup. First, we compute a probabilistic forecast of the population of Norway by age and sex, but irrespective of migration background, using Alho’s Model for Scaled Error (Alho, 1998; Alho & Spencer, 2005). The development of the population to 2060 is simulated 3 000 times by stochastically varying parameters for mortality, fertility and international migration. Next, using annual data for immigrants and their children for 2000–2021, we compute their age- and sex-specific shares relative to the whole population. We use relational models for the age patterns in these shares, and time series models to extrapolate the parameters of the age patterns. We add migrant group detail to the probabilistic forecast using stochastically varying predictions from 3 000 simulations for the shares. This results in a probabilistic forecast for six population sub-groups with immigration background, and one for the non-immigrants. We calibrate the probabilistic forecast against the Medium Variant of Statistics Norway’s official population projection.

## Immigrant population: definitions and issues

Whether a person is counted as an immigrant can be defined in several ways, and different definitions lead to different statistics. One could use rules based on nationality, on ethnicity, on having migrated to a different country, or simply on country of birth. Nationality is problematic, because persons may change nationality after migration. Thus, someone who used to be considered as an immigrant, becomes a non-immigrant simply as the result of a legal procedure. Ethnicity is problematic, because the issue can be sensitive and subjective, and difficult to define (Jacobs et al., 2009). A simple rule is to consider as an immigrant anyone born outside the country. However, a child of native parents who temporarily resided abroad will be labelled as immigrant, and this is not useful in many cultural studies of migrants. Therefore, a narrower definition restricts immigrants to persons born abroad with one or two foreign-born parents. Statistics Norway adds further restrictions for the number of grand-parents who were born abroad, see below. These types of restrictions are also helpful in case one defines the notion of “second generation”. One possibility is to consider a person as second generation when he or she is born in the country with at least one parent and at least two grand-parents born abroad. Rules of this kind help to solve definitional problems in cases where one parent is an immigrant (“first generation”), whereas the other parent is not.

We adopted the definition of immigrant used by Statistics Norway: see https://www.ssb.no/en/befolkning/innvandrere/statistikk/innvandrere-og-norskfodte-med-innvandrerforeldre. An immigrant is a person legally residing in Norway, who was born abroad to two foreign-born parents and four foreign-born grand-parents. This definition does not in itself suggest any racial or cultural connotation—the criterion is place of birth of the parents and of grand-parents. Of the 5.4 million persons who were registered in Norway on 1 January 2022, 898 000 persons were born abroad. Among these, 819 000 were immigrants according to this definition. The definition implies that a refugee or an asylum seeker is not counted as an immigrant until his or her application has been granted. Statistics Norway does not use the notion “second generation” but speaks instead of “Norwegian-born children with two immigrant parents”. Immigrants and their Norwegian-born children together are denoted as “persons with immigrant background”. The definition for children implies that a child with one immigrant and one native parent is not a person with immigrant background.

Immigrants and their Norwegian-born children are classified according to country of origin. For immigrants this is the country of birth. For Norwegian-born children of immigrants, this is the parents' country of birth. If the parents are born in different countries, it is the mother's country of birth.

We have adopted the three country groups that Statistics Norway used in its population projection. Country group 1 comprises all the Western European countries, i.e. countries that were part of the ‘old’ (pre-2004) European Union (EU) and/or the European Free Trade Association (EFTA), as well as the US, Canada, Australia and New Zealand. Country group 2 comprises the eleven new EU countries in Central and Eastern Europe (EU members in 2004 or later): Bulgaria, Croatia,^1^ the Czech Republic, Estonia, Hungary, Latvia, Lithuania, Poland, Romania, Slovakia, and Slovenia. Country group 3 comprises ‘the rest of the world’, e.g., the rest of Eastern Europe, Africa, Asia (including Turkey), South and Central America and Oceania (excluding Australia and New Zealand). See Thomas and Tømmerås (2022 p. 37, p. 153) for details.

In Norway, the population statistics are based on the National Population Register. One problem is that many persons leave Norway, but do not notify the authorities (Vassenden, 2015). This means that they remain recorded in the population register as legal residents. The National Population Register has procedures for adjusting the status of persons who no longer reside in Norway (“administrative deregistration”). For the period 2004–2013, this concerned 26 per cent of all emigrations (Vassenden, 2015). In 2019, however, there was a marked decline in the number of administrative deregistrations of individuals (Thomas & Tømmerås, 2022, p. 102). These administrative procedures imply that statistics about immigrant stocks may lag behind actual developments, and that numbers are a few per cent too high.

## Statistics Norway’s projection

Statistics Norway has a long history of producing the official population projections for Norway, which goes back to at least 1969; see Texmon (1992). For many of the previous projections, future population trends were broken down by age, sex, and municipality of residence. However, as of the projections published in 2005, results for immigrant stocks were also included.

The most recent national population projections were published in July 2022; see Thomas and Tømmerås (2022).^2^ That report gives results on future trends in fertility, mortality, immigration, and emigration, as well as population pyramids for the years 2022–2100. Immigrants from three country groups, their Norwegian-born children, and the rest of the population were projected as separate groups. More detailed information is available from Statistics Norway’s data base “StatBank”; www.ssb.no/en/befolkning/befolkningsframskrivinger/statistikk/nasjonale-befolkningsframskrivinger.

Different scenarios are provided for future fertility, life expectancy, and immigration. For each of these components, three different scenarios were created, labelled as High, Medium, and Low.^3^ The Medium Variant of the projections, considered as the most plausible and labelled as “MMM”, is based on a combination of medium fertility, medium life expectancy, and medium immigration. Relatively strong population growth (“HHH”) results from combining high fertility assumptions with high life expectancy and high immigration, and low population growth (“LLL”) is based on low assumptions for each of the three components. The Medium Variant projects a population size that grows from the current 5.4 million to 6.1 million in 2060 and 6.2 million in 2100. Population ageing continues: the share of persons aged 70 or more, which was around 6 per cent in 1950, is expected to increase further from today’s 13 per cent today to 22 per cent in 2060 and 25 per cent in 2100. The number of young people (0–19) will remain constant. By 2060, they will be outnumbered by the population aged 70 + . The Medium Variant also expects an increasing number of immigrants, growing from 819 000 today to 1.18 million in 2060. The number of Norwegian-born to two immigrants is likely to more than double: 206 000 today and 437 000 in 2060.

## Brief outline of the method

The first step consisted of stochastic simulations of a forecast model of the cohort component type for the population of Norway during the years 2022–2100. This stochastic population forecast is based on Alho’s Model for Scaled Error (Alho & Spencer, 2005). It updates a similar forecast for Norway published in 2020 (Keilman 2020; see also Foss, 2012). We replaced the jump-off population of the previous stochastic forecast by the registered population broken down by age and sex as of 1 January 2022. Age- and sex-specific rates and numbers for fertility, mortality, and net migration from the Medium Variant of Statistics Norway’s, 2022 national projections served as point predictions for the updated stochastic forecast. Finally, uncertainty parameters for fertility, mortality, and net migration, i.e., variances for vital rates and migration numbers, as well as (auto-)correlations between these rates and numbers, were taken from the previous stochastic forecast. We assumed relatively large variances for vital rates and migration numbers for the years 2022—2026, due to uncertainty about the effects of the Covid-19 pandemic and the war in Ukraine. See Keilman (2020) for details. The development of the population to 2060 was simulated 3 000 times by stochastically varying parameters for mortality, fertility, and international migration.

Next, we added migrant group detail to the stochastic population forecast. Each simulated population number for a given age, sex, at a certain future year, was broken down into nine population subgroups according to immigration background (as defined in Sect. 2), using shares that were randomly chosen from their assumed predictive distributions. We modelled the distributions for the migrant group shares, disaggregated by sex and one-year age group. Each share, for a given year, age, sex, and migrant group, has an assumed normal probability distribution in the logit scale. This distribution was calibrated against the Medium Variant of Statistics Norway’s, 2022-based projection (Thomas & Tømmerås, 2022). The result was a set of 3 000 trajectories for the population of Norway broken down by age, sex, and migrant group, for selected years: 2030, 2040, 2050, and 2060.

**Fig. 1:**
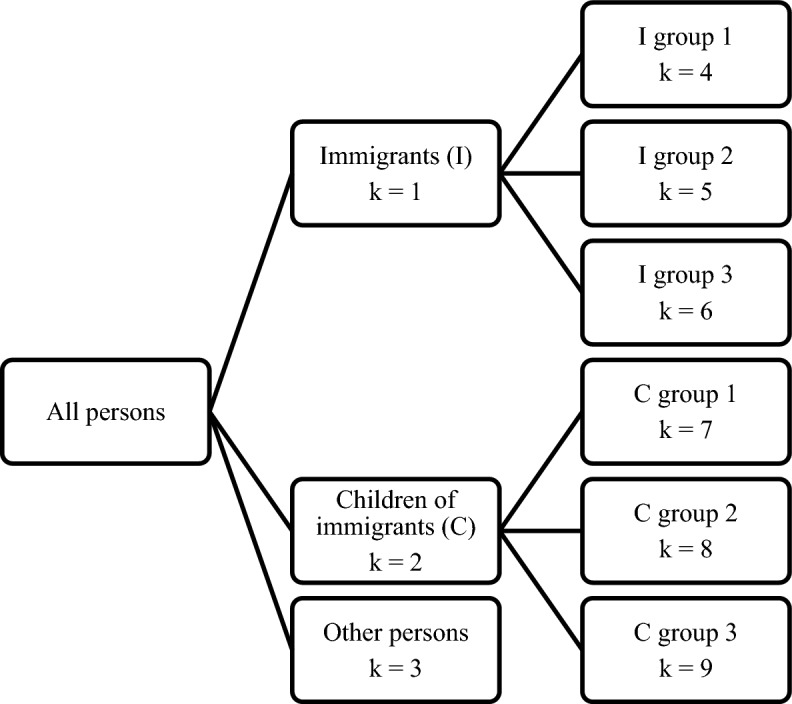
Tree-like structure of persons with immigration background. “Immigrants”, “Children of immigrants”, and country groups 1, 2, and 3 as defined in Sect. 2

We have adopted a tree-like structure when modelling the shares, dividing the population (given age and sex) into three groups: immigrants, Norwegian-born children of immigrants, and the rest of the population. Both the immigrants and their children were divided further into three country groups. This gave six groups of persons with an immigration background, in addition to the remaining part of the population (“population without immigration background”, cf. Section 2). Figure 1 shows the tree-like structure.

We modelled the shares for immigrants and their children. To model the shares for the group of other persons is not necessary, because the three shares sum to one. Similarly, we modelled two of the three shares for immigrants and two of the three shares for the children of immigrants. For each group and for both sexes, we have a table with observed values of the shares for the years 2000–2021, and ages 0–100. We assumed that the shares in each table can be written as a function of time and age. We extrapolated the function into the future and simulated predictive distributions for the extrapolated shares.

## Random shares

We write V(k,x,s,t) for the number of people in migrant group k = 1, 2,…,9 who are at age x = 0, 1,… and are of sex s = 1 or 2, at time t = 0, 1, 2,…. The sum ∑_k_V(k,x,s,t) gives the population W(x,s,t) of age x and sex s at time t, irrespective of migrant group. Migrant group k has share α(k,x,s,t) = V(k,x,s,t)/W(x,s,t) = α_k_(x,s,t) in the population of age x and sex s at time t. The migrant groups are numbered as follows (cf. Figure 1): immigrants (k = 1), Norwegian-born children of immigrants (k = 2), other persons (k = 3), immigrants from country groups 1, 2, and 3 (k = 4, 5, and 6, respectively), and immigrants’ children from country groups 1, 2, and 3 (k = 7, 8, and 9). Often, we will denote the various groups of interest by the following obvious codes: I for immigrants (k = 1), C for children of immigrants (k = 2), O for other persons (k = 3), I1, I2, and I3 for immigrants from country groups 1, 2, and 3 (k = 4, 5, and 6 respectively), and C1, C2, and C3 for immigrants’ children from country groups 1, 2, and 3 (k = 7, 8, and 9).

For a given migrant group, year, and sex, we model the age profiles, in other words, the shares α_k_(x,s,t) as a function of age. These age profiles are specified by means of a few parameters. The parameters may vary over time for men and women who belong to a certain migrant group. The focus is on finding appropriate functions for the age profiles, and appropriate time series models for their parameters.

### Descriptive analysis for the period 2000–2021

We have used annual data from Statistics Norway for the period 2000–2021 (1 January) on persons who have legal residence in Norway, broken down by sex, age (0, 1, …, 100), and migrant group (k = 1–9).

**Fig. 2:**
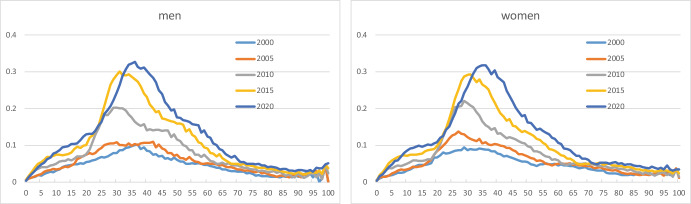
Age-specific shares of immigrants (k = 1) for men and women aged 0–100, selected years

Fig. 2 plots age profiles for the shares of immigrants α_1_(x,s,t) for men and women aged 0–100 years for selected calendar years. The shares are aggregates over country groups. Many immigrants are aged 20–60. The age profiles are very similar for men and women. The shares of immigrants increase sharply after 2005, when new member countries joined the EU, and immigration from these countries to Norway became easier. However, immigrants from group 3 countries contribute to this increase, too; see below. The curves for 2010, 2015, and 2020 move systematically to higher ages over time, which suggests a cohort effect in the age profiles. Note that all age groups, including the youngest, concern persons born abroad. Age groups below 20, say, are children who immigrated, alone or together with one or both parents, or who came to Norway after adoption.

**Fig. 3:**
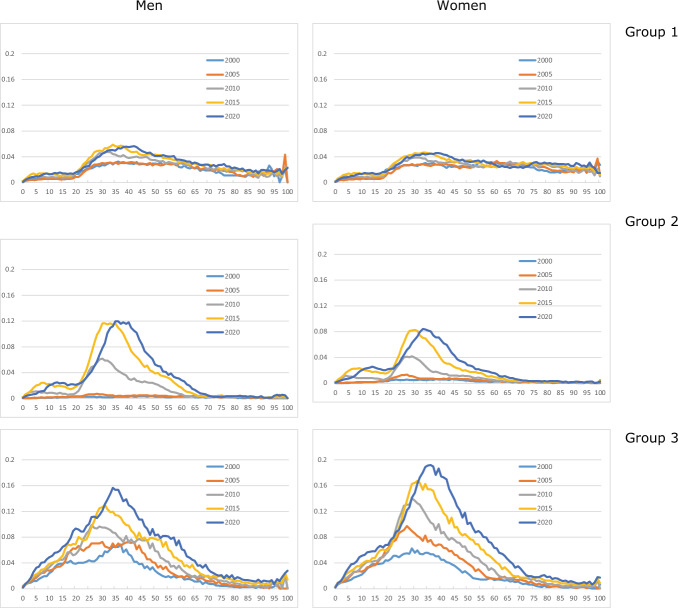
Age-specific shares of immigrants from country groups 1 (k = 4), 2 (k = 5), and 3 (k = 6), for men and women aged 0–100, selected years

Fig. 3 shows shares for immigrants from the three country groups. Group 1 immigrants are less prevalent than those from elsewhere. Children and young adults have low shares for country groups 1 and 2, compared to group 3: many of the group 1 and 2 immigrants come as labour immigrants and stay for a limited period, whereas many group 3 immigrants have a background as refugee or asylum seeker, and family reunification is relatively frequent.

Immigration from new EU member countries in Central and Eastern Europe increased considerably after the enlargement of the EU in 2004; see country group 2. However, immigrant shares for Western countries (group 1) were rising slightly as well in this period, caused by peak immigration flows in the years 2007–2015. The curves for the remaining part of the world increase regularly. For a given country group, the age profiles show similar shapes for men and women. Labour migration could be a factor that explains why men in group 2 have somewhat higher shares in recent years than women.

**Fig. 4:**
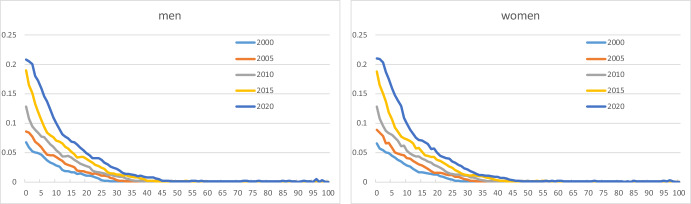
Age-specific shares of Norwegian-born children of immigrants (k = 2) for men and women aged 0–100, selected years

Figure 4 illustrates the share profiles of Norwegian-born children of immigrants irrespective of country group. The curves are very similar for boys/men and girls/women. The profiles increase regularly over time. Shares beyond age 50 are very small. This reflects the fact that many of the immigrants came to Norway only a few decades ago, and hence their children who were born in Norway are relatively young.

**Fig. 5:**
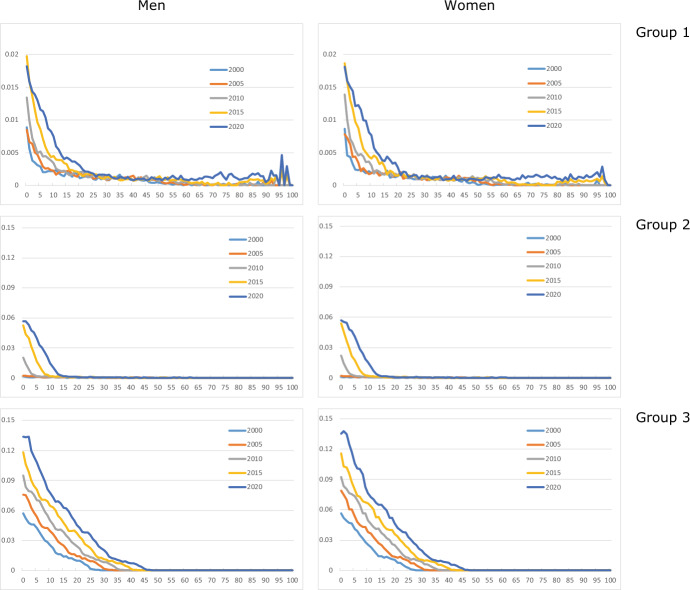
Age-specific shares of Norwegian-born children of immigrants from country groups 1 (k = 7), 2 (k = 8), and 3 (k = 9), for men and women aged 0–100, selected years. Note: vertical scales differ between country groups

Most of the children of immigrants belong to country group 3 (Fig. 5). In all cases, we observe a more or less regular rise over time in the age profiles. For children from group 2, the increase did not start until around 2005, after the enlargement of the EU.

### Modelling the shares

To ensure that predicted shares are within the [0, 1] interval, we have used a multinomial logit transformation. For immigrants (k = 1), Norwegian-born children (k = 2), and other persons (k = 3) and a given year t, age x, and sex s, define the transformed shares as

$$\beta _{1} = \ln \left( {\frac{{\alpha _{1} }}{{\alpha _{3} }}} \right)\;{\text{and}}\;\beta _{2} = \ln \left( {\frac{{\alpha _{2} }}{{\alpha _{3} }}} \right),\;\alpha _{1} + \alpha _{2} + \alpha _{3} = 1.$$

The population subgroup “other” (k = 3) is arbitrarily selected as the benchmark.

A second transformation defines country group-specific shares of immigrants in the logarithmic scale:

$$\beta_{4} = \ln \left( {\frac{{\alpha_{4} }}{{\alpha_{6} }}} \right)\;{\text{and}}\;\beta_{5} = \ln \left( {\frac{{\alpha_{5} }}{{\alpha_{6} }}} \right),\; \alpha_{4} + \alpha_{5} + \alpha_{6} = \alpha_{1} ,$$

Using immigrants from country group 3 (k = 6) as the benchmark group.

Finally, define transformed shares for Norwegian-born children of immigrants from country groups 1 (k = 7) and 2 (k = 8) as:

$$\beta_{7} = \ln \left( {\frac{{\alpha_{7} }}{{\alpha_{9} }}} \right)\;{\text{and}}\;\beta_{8} = \ln \left( {\frac{{\alpha_{8} }}{{\alpha_{9} }}} \right),\; \alpha_{7} + \alpha_{8} + \alpha_{9} = \alpha_{2} ,$$

where children from country group 3 (k = 9) are the benchmark group. The second and third transformations are examples of a generalized logit transformation (Mead, 1965). For brevity, we will use the term logit transformation throughout for all β’s.

The result of the logit transformation of shares is six sets of β’s for immigrants and children, broken down by age (0–100 years), sex (men and women), and calendar year (2000–2021). This means that we have a total of 6 × 101 × 2 × 22 = 26 664 β-values, or 4 446 for each migrant group.

We assume that each β is normally distributed, with mean and variance that may depend on k, x, s, and t. The challenge is to predict them to future years, and to find the variances of the prediction errors. The predictions themselves follow from the Medium Variant of Statistics Norway’s official forecast.

Initially, we have assumed

1 $$\beta_{k} \left( {x,\;s,\;t} \right) = a_{k} \left( t \right) + b_{k} \left( {x,\;s} \right) + e_{k} \left( {x,\;s,\;t} \right),\;k = 1,\;2,\;4,\;5,\;7,\;8$$

The function b_k_(x,s) is commonly known as the *standard age profile* and the model describes how β in a certain year differs from the standard. This so-called relational approach has been used in the context of mortality (Brass, 1971; De Beer, 2012), fertility (Booth, 1984; De Beer, 2011; Zeng et al., 2000), and nuptiality (Coale & Trussell, 1974). The well-known Brass relational model is a special case of model (1), namely one for a fixed time t. Originally intended for modelling age-specific survival, it can be written as Y(x) = a + b・Y^S^(x) + e(x). Y(x) is the logit-transformed probability of survival from birth to age x, while Y^S^(x) is some standard age pattern of survival, also in logit form. a and b are coefficients to be estimated from the data, and e(x) is an error term. Changing parameter a shifts the age pattern up or down relative to the standard, while b changes its slope. See e.g. Preston et al., (2001, pp. 199–201).

To allow the maximum of flexibility, we adopted a non-parametric approach, and specified both a_k_(t) and b_k_(x,s) in expression (1) as a sum of terms, one for each year t (t = 2000, 2001, …, 2021) and one for each age x (x = 0, 1, …, 100). In addition, we assumed different age profiles for men compared to women. For a given migrant group, we assumed

2 $$\beta \left( {x,\;s,\;t} \right) = \sum\limits_{{i = 2000}}^{{2021}} {a_{i} 1_{i} \left( t \right)} + \sum\limits_{{i = 0}}^{{100}} {b_{{i,s}} 1_{i} \left( {x,\;s} \right) + e(x,\;s,\;t)}$$

The indicator function 1_i_(j) equals 1 for i = j, and 0 otherwise. Coefficients a_t_ and b_x,s_ are to be estimated from the data; they represent the time effects and the age effects, respectively, of the array β(x,s,t). For instance, for immigrants from country group 1, we found a positive trend in the coefficients a_t_ (t = 2000, 2001, …, 2021), implying that this migrant group has become more prevalent, compared to the immigrants from country group 3 (the reference group).

Model (2) contains many parameters. In order to reduce the risk of overfitting, we estimated a more parsimonious model. The α-shares relate to stocks of persons. Therefore, they tend to change slowly over time (with some exceptions). The same is true for the β’s. Time effects for the various groups showed very regular upward or downward trends, with two exceptions (immigrants and children from country group 2; see Sect. 5.3). A special situation occurs when the time effect is a linear function of time. In that case model (2) becomes

3 $$\beta \left( {x,\;s,\;t} \right) = A_{0} + B_{1} t + \sum\limits_{i = 0}^{100} {b_{i} 1_{i,s} \left( {x,\;s} \right) + e\left( {x,\;s,\;t} \right)}$$

with a first difference of β of

4 $$\Delta \beta \left( {x,\;s,\;t} \right) = \beta \left( {x,\;s,\;t} \right) - \beta \left( {x,\;s,\;t - 1} \right) = B_{1} + d\left( {x,\;s,\;t} \right)$$

where d(x,s,t) = e(x,s,t) – e(x,s,t–1). For the moment, we assume that d(x,s,t) is uncorrelated across time, but will come back to this issue towards the end of Sect. 5.3, and in the online appendix. Model (4) represents a Random Walk with Drift (RWD). The time-increment in each β of a given age equals a constant value (“drift”) plus a random term. However, it is unlikely that the time-increments are the same for each age. A more flexible model is

5 $$\Delta \beta \left( {x,\;s,\;t} \right) = A_{1} + B_{1} \cdot \beta ^{S} \left( {x,\;s} \right) + d\left( {x,\;s,\;t} \right)$$

where β^S^(x,s) is a standard age pattern in the spirit of the Brass model, to be defined below. Note that model (5) for the increments Δβ(x,s,t) is consistent with a model for β(x,s,t) that includes an interaction between time and age (in addition to a linear time effect).

### Model estimates

**Fig. 6:**
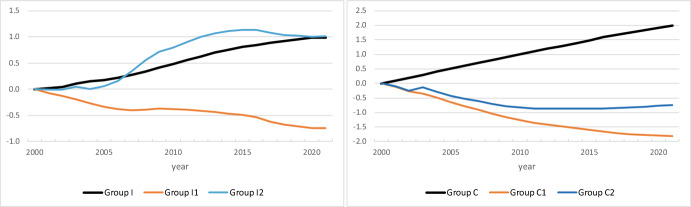
Time effects a_t_. Year 2000 is reference year (a_2000_ = 0)

**Fig. 7:**
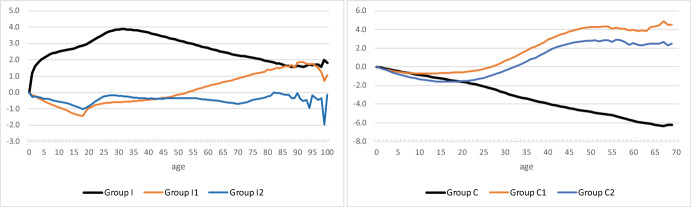
Age effects b_x_, for men and women combined. Age 0 is reference age (b_0_ = 0)

Estimation of the parameters of model (5) was done in two steps. First, we computed the time effects a_t_ and age effects b_x,s_ in model (2) for each group by taking age-averages $\sum\nolimits_{x} {\beta (x,\;s,\;t)/101}$ and time-averages $\sum\nolimits_{t} {\beta (x,\;s,\;t)/22}$, respectively, of observed *β*-values.^4^ Results for men and women were very close. Hence, Figs. 6 and 7 show time effects and age effects for the two sexes combined. The age effects were irregular at high ages, due to the small numbers involved, in particular for Groups C, C1, and C2. Therefore, we restricted computation of age effects for the latter three groups to ages below 70. When interpreting the results, one should keep in mind that for the group in question, the results are relative to both a reference year (year 2000; Fig. 6) or reference age (age 0; Fig. 7), *and* the share in the benchmark group. As an example, take time effects for immigrants irrespective of country group (group I) in Fig. 6. Across all ages 0–100, β-values (“prevalence”) for persons in this group increase faster than did the values for members of the benchmark group “other” (k = 3). Indeed, international migration accounted for 62 per cent of population growth during the years 2000–2021 (Statistics Norway, 2022). Country group 2 includes 10 countries in Central and Eastern Europe that joined the EU in 2004, and two countries that became members in 2007. This explains the steep increase in the time effect for immigrants from these countries (group I2). The curve flattens out around 2015 at the time of the Syrian refugee crisis, implying that reference group I3 became more prevalent. Figure 6 shows also that immigrants from group 3 (compared to immigrants from country group 1) and their children became more prevalent during the period 2000–2021, as reflected in falling trends in time effects for groups I1, C1, and C2. Except for migrants and children from country group 2, the trends are very regular.

Age effects are more irregular and difficult to interpret. For example, take immigrants irrespective of country group (group I) in Fig. 7. Across all years 2000–2021, their *β*-values (“prevalence” compared to the benchmark group) are much larger for adult than for young ages. Indeed, from Figs. 2 and 4 we can conclude that across all years, β is roughly equal to ln(0.01/(1–0.01–0.1)) =  − 4.5 at age 0, but that it is approximately ln(0.25/(1–0.25–0)) =  − 1.1 at age 35. When we shift the curve with the age effects upwards, such that the effect is zero for the reference persons of age 0, we find large positive age effects for adults in this group. Age patterns for Norwegian-born children of immigrants (group C; k = 2) are very regular, as noted before.

Given the regular time trends in estimated time effects, in a second step we adopted model (5) as a good representation of β_k_(x,s,t) for groups I, I1, C, and C1 for the period 2000–2021. For groups I2 and C2, a more or less linear time effect since 2015 seems to be a better basis for extrapolation into the future. For each group, we used for the age profile β^s^(x) the mean of observations $\sum\nolimits_{t} {\beta (x,\;t)/22}$.

Table 1 shows results irrespective of sex, as the estimates differed very little between men and women. In addition, the table gives estimates of the covariance between the estimators of the two parameters, and of the standard deviation of the error terms d(x,s,t), to be used later.

**Table 1:** Parameter estimates for model (5). Data for the years 2000–2021 (groups I, I1, C, and C1); for the years 2015–2021 for groups I2 and C2. Men and women combined. Student *t*-values based on robust standard errors

Group	k	A_1_		B_1_		cov(A_1_,B_1_)	σ_k_
		Estimate	*t* value	Estimate	*t* value		
I	1	0.094	12.5	0.017	5.5	0.0231E − 3	0.112
I1	4	− 0.042	− 10.5	− 0.021	− 4.9	0.0140E − 3	0.190
I2	5	− 0.146	− 6.9	− 0.087	− 6.5	0.2776E − 3	0.133
C	2	0.066	6.0	− 0.006	− 2.0	0.0335E − 3	0.214
C1	7	− 0.218	− 8.7	− 0.072	− 6.8	0.259E − 3	0.319
C2	8	− 0.262	− 4.0	− 0.107	− 4.9	1.3910E − 3	0.360

The estimates of A_1_ and B_1_ are difficult to interpret but note that all have high *t*-values. Yet the proportion of variance explained by the model (not shown in the table) is very low, typically eight per cent or less. For all six groups the residuals, when plotted in a histogram, show a very symmetric shape, although a qq-plot indicates heavier tails than a normal distribution would imply. In order to check the robustness of our findings, we analysed a number of variants of model (5). The online appendix for this paper reports results for a model that (1) includes a cohort effect; deals with (2) autocorrelation and with (3) both autocorrelation and heteroscedasticity in the error term d(x,s,t); (4) includes a quadratic term of the age profile; (5) was estimated using shorter time series of data (years 2002–2021 and years 2000–2019). These alternative versions of model (5) gave results that were very close to those in Table 1.

### Predicted shares

Starting from a known value β(x,s,T) for a given group k, a future value h years ahead (h = 1, 2, …) is

6 $$\beta \left( {x,\;s,\;T + h} \right) = \beta \left( {x,\;s,\;T} \right) + h.\left( {A_{1} + B_{1} .\beta^{S} \left( x \right)} \right) + \mathop \sum \limits_{j = 1}^{h} d(x,\;s,\;T + j)$$

The h-step ahead forecast E[β(x,s,T + h)] is β(x,s,T) + h. (${\widehat{\text{A}}}_{1}$+${\widehat{\text{B}}}_{1}$. β^S^(x)), where we have replaced A_1_ and B_1_ by their estimates. The forecast error F(x,s,T + h) equals β(x,s,T + h)–E[β(x,s,T + h)]. Given our assumptions, its variance Var[F(x,s,T + h)] can be estimated as

7 $$ \begin{gathered} Var\left[ { \mathop \sum \limits_{i = 1}^{h} d\left( {x,T + i} \right) - h \cdot\left( {\hat{A}_{1} + \hat{B}_{1} \cdot\beta^{S} \left( x \right)} \right)} \right] \hfill \\ \;\;\;\;\;\; = h \cdot \hat{\sigma }_{s}^{2} + h^{2} \cdot Var \left[ {\hat{A}_{s} } \right] + h^{2} \cdot\left( { \beta^{S} \left( x \right)} \right)^{2} Var\left[ {\hat{B}_{1} } \right] - 2\cdot h \cdot \beta^{S} \left( x \right)\cdot Cov \left[ {\hat{A}_{1} ,\hat{B}_{1} } \right], \hfill \\ \end{gathered} $$

where $\sigma_{s}^{2}$ is the variance, for a given group k, of the error term d_k_(x,s,t) of model (5).

#### Correlations

When predicting logit-shares β_k_(x,s,t), one has to take into account possible correlations across ages, sexes, and migrant groups. Since we model each β as a Random Walk with Drift, it has zero autocorrelation. We estimated correlations across migrant groups, ages, and between men and women from the residuals of model (5).

The residuals for six migrant groups have (6 × 5)/2 = 15 pairwise correlations. Of these, eight were negative, seven were positive. Thirteen correlation estimates turned out to be moderate or low: between − 0.265 and + 0.125. Eight estimates are not significantly different from zero at the five per cent level. Quite strong correlations are those between I1 and I2 (0.519), and between C1 and C2 (+ 0.337). The mean and the median values of the fifteen correlations are 0.019 and − 0.0075, respectively. There is no clear pattern in the fifteen estimates: some are positive, others are negative-most correlations are modest or small, two of them are large. Since these results are hard to interpret, we have assumed that migrant groups are uncorrelated.

Table 2 shows pairwise correlations between men and women. They are higher for migrant groups I, I1, and I2 than for children of groups C, C1, and C2. One explanation is the following. The correlations derive from the residuals of model (5), which describes first differences in β-transformed shares. Since the shares reflect stocks, their first differences derive from changes in stocks. For migrant groups I, I1, and I2, the larger part of the changes stems from immigration—mortality plays a minor role, because the migrants are relatively young. At the macro level, immigration for these groups is positively correlated between men and women. Shares for children groups C, C1, and C2 change due to mortality and outmigration (and fertility for age 0), because all children are born in Norway. In this case the numbers involved are much smaller, and hence the changes are more volatile and less systematic than changes caused by immigration for groups I, I1, and I2. In the simulations, we used average correlations for groups I, I1, and I2 (0.4869) and groups C, C1, and C2 (0.1725).

**Table 2:** Correlations between men and women, by migrant group

I	I1	I2	C	C1	C2
0.4681	0.5337	0.4589	0.0290	0.1685	0.3200

Errors are possibly correlated across ages. We assumed a first-order auto-regression (AR1) process for the errors (e.g., Alho & Keilman, 2010; Christiansen & Keilman, 2013). Table 3 gives estimated correlations by migrant group.

**Table 3:** Correlations across ages, by migrant group

I	I1	I2	C	C1	C2
− 0.2032	− 0.2876	0.1219	− 0.1769	− 0.2095	− 0.2810

Five out of six migrant groups show estimates around − 0.2. The negative values are surprising. They suggest that when a β-value for a certain age x is larger than expected, the values for neighbouring ages (x − 1) and (x + 1) are smaller than expected. The reason for this finding is unclear, but for groups I, I1, and I2 it might be associated with the volatility of annual migration flows. Note, however, that all correlations Corr[d(x,t),d(x + 1,t)] are computed period-wise. As mentioned earlier, a cohort effect is visible in the shares for many groups. Indeed, *cohort-wise* correlations Corr[d(x,t),d(x + 1,t + 1)] turned out to be positive and strong, around 0.8 for groups I, I1, and I2, and 0.95–0.99 for groups C, C1, and C2. Since the results in Table 3 are difficult to interpret, and the values are modest to small, we have assumed that the *β*-values are uncorrelated across ages in a given future year, given sex and migrant group.

#### Predictions

**Fig. 8:**
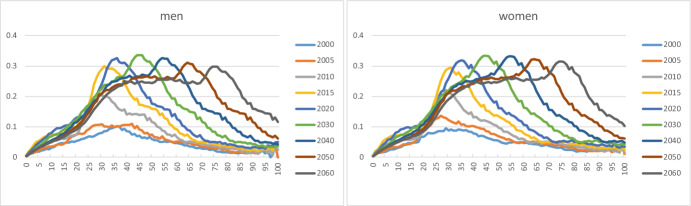
Age-specific shares of immigrants (k = 1) for men and women aged 0–100, selected years

**Fig. 9:**
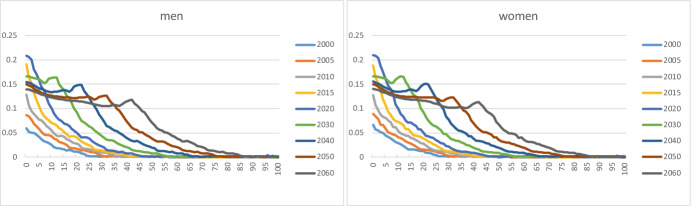
Age-specific shares of Norwegian-born children of immigrants (k = 2) for men and women aged 0–100, selected years

We used Statistics Norway’s projection results for 2030, 2040, 2050 and 2060 to compute shares α_k_(x,s,t) for these years. Figures 8 and 9 extend the shares α_k_(x,s,t) for groups I and C in Figs. 2 and 4 with future values.

Men and women in group I born around 1985–1990 show large shares (Fig. 8). Statistics Norway predicts strong ageing for these persons. The shares of younger cohorts level off at around 25 per cent as soon as they reach adult ages. As to children (Group C in Fig. 9), a new pattern seems to emerge after 2020. Historical curves show a regular decline with age and an increase over time. The latter increase disappears for future years, whereas the decline in the age direction becomes a bit irregular. The explanation lies in the shares of children of group C3, who make up by far the largest shares in group C (cf. Figure 5). The fertility of immigrant women from Asia and Africa, who form a major sub-group of mothers of C3-children, has systematically declined during the years 2011–2021, with a particularly strong drop starting in 2017.^5^ Statistics Norway has extrapolated this decrease to future years, which results in age patterns of shares that decline over time. The small peaks in the age patterns for given years reflect the large number of C3-children born in the years 2014–2016.

Target values of the shares α_k_(x,s,t) for the years 2030, 2040, 2050, and 2060 were transformed into β_k_(x,s,t)-values, using the expressions of Sect. 5.2 "Modelling the shares". The latter values served as expected values for the predictive distributions of β_k_(x,s,t). The variances of β_k_(x,s,t) follow from expression (7), and we assumed normality, as stated before. The distributions were simulated based on N = 3 000 random draws for each of the four future years, and each combination of k, x, and s.

Expressions for the back-transformation from β_k_(x,s,t) to α_k_(x,s,t) are (temporarily suppressing x, s, and t),

8 $$ \begin{gathered} \alpha_{1} = \frac{{\exp \left( {\beta_{1} } \right)}}{{1 + \exp \left( {\beta_{1} } \right) + \exp \left( {\beta_{2} } \right)}},\;\alpha_{2} = \frac{{\exp \left( {\beta_{2} } \right)}}{{1 + \exp \left( {\beta_{1} } \right) + \exp \left( {\beta_{2} } \right)}},\;\alpha_{3} = \frac{1}{{1 + \exp \left( {\beta_{1} } \right) + \exp \left( {\beta_{2} } \right)}}, \hfill \\ \alpha_{4} = \frac{{\alpha_{1} .\exp \left( {\beta_{4} } \right)}}{{1 + \exp \left( {\beta_{4} } \right) + \exp \left( {\beta_{5} } \right)}},\;\alpha_{5} = \frac{{\alpha_{1} .\exp \left( {\beta_{5} } \right)}}{{1 + \exp \left( {\beta_{4} } \right) + \exp \left( {\beta_{5} } \right)}},\;\alpha_{6} = \frac{{\alpha_{1} }}{{1 + \exp \left( {\beta_{4} } \right) + \exp \left( {\beta_{5} } \right)}}, \hfill \\ \alpha_{7} = \frac{{\alpha_{2} .\exp \left( {\beta_{7} } \right)}}{{1 + \exp \left( {\beta_{7} } \right) + \exp \left( {\beta_{8} } \right)}},\;\alpha_{8} = \frac{{\alpha_{2} .\exp \left( {\beta_{8} } \right)}}{{1 + \exp \left( {\beta_{7} } \right) + \exp \left( {\beta_{8} } \right)}},\;\alpha_{9} = \frac{{\alpha_{2} }}{{1 + \exp \left( {\beta_{7} } \right) + \exp \left( {\beta_{8} } \right)}} \hfill \\ \end{gathered} $$

More formally, we assumed that for a given combination of k, x, and s, the distribution of β in a future year t is N(μ,σ^2^), where μ is the β-transformed value of the target share α, and σ^2^ follows from expression (7). 3 000 random numbers β^r^ (r = 1, 2,…, 3 000) were drawn from this distribution, taking into account the correlation between men and women. Each β^r^ was transformed to a corresponding α^r^. This resulted in 3 000 simulations for each share α_k_(x,s,t).

For a given migrant group, age, sex, and year, the 3 000 predicted shares α^r^ (r = 1, 2, …, 3000) were multiplied with 3 000 simulated population numbers W^r^ (irrespective of migrant group) from the stochastic population forecast. The result was a set of simulated values V^r^ (r = 1, 2, …, 3 000) for the population by sex (men, women), age (0, 1, 2, …, 99, 100 +), and seven categories of migration background (immigrants and Norwegian born children, both for three country groups, and other persons) for the years 2030, 2040, 2050, and 2060. In many cases the mean of the simulated values (Σ_r_V^r^)/3000 for a certain combination of age, sex, migrant group, and calendar year differed strongly from the corresponding target value V from the official projections. In some cases, the target value was even below the 10-th percentile, or larger than the 90-th percentile, of the set of V^r^-values. The discrepancies were larger for 2050 and 2060 than for 2030 or 2040. The difference between the mean of the simulated V^r^-values and the target value V is caused by the exponential back-transformation (8). The details are complicated, but an approximate argument is as follows. Assume that a random variable X has a normal distribution N(µ,σ^2^). Define a new random variable as Y = exp(X). Y has a log-normal distribution with expected value exp(µ + ½σ^2^), which differs from exp(µ) by a factor exp(½σ^2^). Although the situation in our case is a bit more complicated (a logit transformation and several random variables simultaneously), the argument is similar. The random variable Y above corresponds to α, and X corresponds to β. Each α^r^ is an exponential transformation of a simulated β^r^, yet the mean across all 3 000 α^r^-values differs from the exponentially transformed mean of β^r^-values, which corresponds with the expectation µ. The discrepancy is larger, the larger the variance of the β-estimate is.

Each simulated number V^r^, given age, sex, migrant group, and calendar year was adjusted proportionally with the ratio of the target value V and the mean of the 3 000 simulated values. This led to a mean value across the simulations equal to the target value.

## Results

### Total population

The results for the stochastic forecast show mean predicted population sizes in 2030, 2040, 2050 and 2060 equal to 5.66, 5.89, 6.03 and 6.11 million, respectively. These numbers agree with the results of the Medium Variant of Statistics Norway’s population projection, as expected. The 80 per cent prediction intervals are—in millions—[5.57–5.75], [5.66–6.13], [5.63–6.46], and [5.50–6.77] for these four years. In contrast, results for the Low and the High Variants of the official projection show much wider intervals—for example, [5.18–7.09] million for 2060. The wide intervals are caused by the way Statistics Norway constructed the Variant projections. For example, the High projection Variant assumes that fertility is high in *all* future years, and vice versa for the Low Variant. Similar assumptions apply to the High and Low Variants of life expectancy and of net migration. In contrast, the stochastic forecast for the population by age and sex assumes that fertility, mortality, and net migration have less than perfect autocorrelation. This means that birth rates may be higher than expected in one year, but lower the year thereafter, and similarly for death rates and migration numbers. Moreover, fertility, mortality, and migration are stochastically independent of each other.

**Fig. 10:**
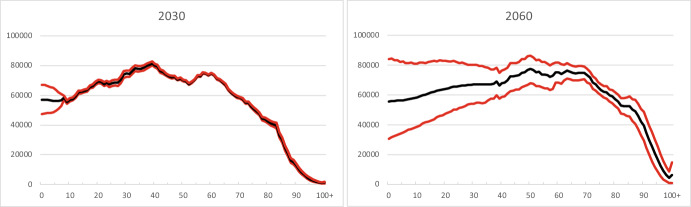
Age distribution, 2030 and 2060. The upper and lower curves are 90 per cent and 10 per cent percentiles (upper and lower bounds of the 80 per cent prediction intervals) of the predictive distribution. The middle curves represent median values

Uncertainty differs strongly between age groups. Prediction intervals are very narrow until roughly 2040, except for children born in the years 2022–2039. This means that forecasts of adults and elderly are rather certain during the first few decades of the forecast period. For later years, uncertainty increases gradually for all age groups. As an illustration, Fig. 10 shows the median value and 80 per cent prediction intervals for the age distribution in 2030 and 2060. Here we will focus on the findings for immigrants and their children.

### Immigrants

Table 4 gives median values, as well as upper and lower bounds of 67 and 80 per cent prediction intervals for the size of the population sub-group of immigrants (irrespective of country group) for selected years between 2030 and 2060.

**Table 4:** Number of immigrants in 2022 (registered), and 2030, 2040, 2050, 2060. Median value, lower and upper bounds of 67 per cent and 80 per cent prediction intervals based on 3000 simulations. Medium Variant and Low and High Variants of Statistics Norway’s projection of 2022

	2022	2030	2040	2050	2060
1000 s					
Median	819	956	1064	1140	1179
67% prediction interval		[920–992]	[1007–1124]	[1065–1219]	[1081–1283]
80% prediction interval		[908–1003]	[988–1143]	[1042–1247]	[1054–1314]
Medium Variant		956	1065	1143	1182
Low–High interval		[902–1034]	[960–1218]	[977–1403]	[949–1585]

The number of immigrants is expected to increase in the next four decades, with a median value in 2060 that is 44 per cent higher than the current 819,000. The lower bounds of the 80 per cent intervals tell us that the increase is almost certain. Chances are 90 per cent that there will be at least 920,000 immigrants in 2030, and 1.054 million in 2060—many more than today. However, we are not at all certain about how steep the increase will be, since the 80 per cent interval for 2060 is rather wide: 22.1 per cent of the median value ((1314 − 1054)/1179). Expressed this way, uncertainty grows regularly from 9.9 per cent in 2030, to 14.6 and 18.0 per cent in 2040 and 2050. At the same time, the interval between Statistics Norway’s Low and High Variants indicates unduly large uncertainty.

**Fig. 11:**
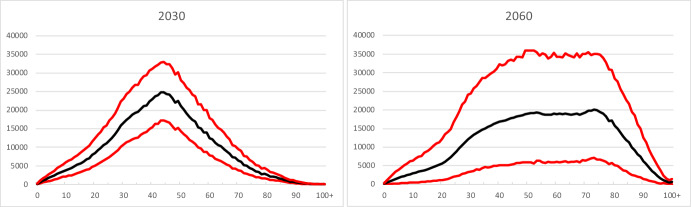
Age distribution of immigrant population, 2030 and 2060. The upper and lower curves are 90 per cent and 10 per cent percentiles (upper and lower bounds of the 80 per cent prediction intervals) of the predictive distribution. The middle curves represent median values

The age distributions in Fig. 11 suggest that predicted numbers for immigrants aged 30–60, say, already in 2030 have so wide prediction intervals that the results for one-year age groups bear little information. By 2060, this is the case for ages between 10 and 90, roughly speaking. In other words, when one needs information about the age structure of immigrants in the future, this can only be in the form of broad age groups in order to be reliable.

The online appendix of this paper reports results for immigrants from groups I1, I2, and I3 separately. For groups I1 and I2, the expected values suggest small increases to 2050, and a stabilization or slight decrease to 2060. However, the 80 per cent prediction intervals are so wide that we cannot be certain this will materialize. The results in the appendix show that the strong increase in the number of immigrants in the future (Table 4) is caused by immigrants from country group 3. Chances are at least 90 per cent that there will be more immigrants in this group in 2060 than today. However, the 80 per cent interval in 2060 is rather wide, which means that we do not know how steep the increase will be.

### Norwegian-born children of immigrants

Statistics Norway projects a strong increase in the number of Norwegian-born children of immigrants. The results in Table 5 confirm this. The median value more than doubles from 2022 to 2060. One can be quite certain about an increase: the lower bound of the 80 per cent interval in 2060 is 350,000, which is 70 per cent higher than today’s number of 206,000 children. Note that the Low–High interval of the official projections agrees quite well with the 80 per cent prediction intervals.

**Table 5:** Number of Norwegian-born children of immigrants in 2022 (registered), and 2030, 2040, 2050, 2060. Median value, lower and upper bounds of 67 per cent and 80 per cent prediction intervals based on 3 000 simulations, Medium Variant and Low and High Variants of Statistics Norway’s projection of 2022

	2022	2030	2040	2050	2060
1000 s					
Median	206	262	328	381	431
67% prediction interval		[244–283]	[293–364]	[335–439]	[367–504]
80% prediction interval		[238–290]	[284–377]	[322–459]	[350–531]
Medium Variant		263	329	387	437
Low–High interval		[247–282]	[285–376]	[314–469]	[337–565]

**Fig. 12:**
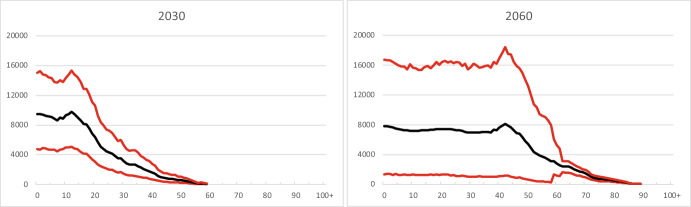
Age distribution of Norwegian-born children of immigrants, 2030 and 2060. The upper and lower curves are 90 per cent and 10 per cent percentiles (upper and lower bounds of the 80 per cent prediction intervals) of the predictive distribution. The middle curves represent median values

Forecast results for Norwegian-born children of immigrants with one-year age group detail (Fig. 12) are not reliable for most ages: ages up to 40 in 2030, and up to 65 in 2060, roughly speaking.

When the group of Norwegian-born children of immigrants is split up by country background, the increases for children in groups 1 and 2 to 2060 are quite reliable; see the online appendix. However, the numbers involved are small, and the 80 per cent intervals in 2060 are very wide. Children who belong to group 3 constitute the large majority of all Norwegian-born children of immigrants. Results in the online appendix indicate more than a doubling between 2022 and 2060 in terms of the median forecast. An increase is very certain, generally speaking.

## Discussion

An alternative to the random share approach is to construct a probabilistic multi-state model for the population broken down by age, sex, and migrant group. In that case one deals with three or four components of change for each migrant group: fertility, mortality, and gross or net flows of international migration. Members of the migrant groups cannot move between groups, because we define migrant group membership by country of birth. Still, a full-fledged multistate model requires that one specifies the predictive distribution for each of the three or four components of change for each migrant group, including correlations across age, sex, and time. To estimate the parameters from available data is a formidable challenge, even in the case of Norway, where register data of good quality are available. Our random shares approach reduces the complexity of the problem to one third or one fourth that of a multistate approach.

We define an immigrant as a person legally residing in Norway, who was born abroad and who has two foreign-born parents and four foreign-born grandparents. Immigrants represent one population sub-group in our approach. Norwegian-born children of two immigrant parents comprise a second sub-population. The remaining group is very heterogeneous. It consists not only of the native population, but also of persons born abroad with at most one foreign-born parent, and/or fewer than four foreign-born grandparents. Norwegian-born children with only one immigrant parent belong to this third sub-population, too. One could refine the present model and introduce separate categories for persons with a mixed background. Statistics Norway publishes some data for these persons (https://www.ssb.no/en/statbank/table/12548/), but age details are lacking.

We have modelled each share as a Random Walk with Drift. An alternative strategy, as one reviewer pointed out, could be to start from expression (3) and build a variance-component model for the error term e(x,s,t,k). For instance, one could distinguish one component for errors e_1_(t,k) by time and migrant group only, and a second component e_2_(x,s,t,k) for the full breakdown, where e_1_ and e_2_ would be independent of each other, and over time. Data analysis can tell if there are correlations across cohort lines in e_2_, or if there is an age effect in e_1_. While this is certainly a viable strategy, our model includes a possible cohort effect directly: expression (5) is consistent with a model that captures such cohort effects. Furthermore, β^S^(x,s) in (5) represents age effects already.

The multiplication α^r^.W^r^ = V^r^ implicitly assumes that the random variables α and W are uncorrelated, an assumption that one may criticize. For example, if the count of 60-year olds in 2060 is higher (lower) than expected, this might be due to higher (lower) numbers of immigrants into this cohort 25–40 years earlier. This would translate into a higher (lower) share of immigrants in 2060 than expected, and a positive correlation between counts and shares. However, the large (small) count in 2060 might also be due to high (low) birth rates 60 years earlier. Fertility, mortality, and migration are assumed independent in the stochastic forecast for counts W^r^, following usual practice (Alho & Spencer, 2005). Hence it is reasonable to assume that the effects of possible correlations between shares α^r^ and population counts W^r^ are small enough to be ignored.

## Conclusions

No forecasts are exact, so it is important to provide some measure of the forecast uncertainty. Therefore, forecasters should compute two types of results: first, point forecasts, which are as accurate as possible, and second, the statistical distributions around the point forecasts (Makridakis et al., 2019).

In July 2022, Statistics Norway published a deterministic population projection for the migrant population of Norway and their children. Based on data for the years 2000–2021, we add statistical distributions around forecasts of the size and age and sex structure of these sub-populations. We use the method of random shares, which starts with a stochastic forecast of the future population broken down by age and sex. Each result of the latter forecast is the outcome of a random variable. This variable is combined with a set of random shares that divide each future population number, given age and sex, into numbers for migrant categories. We present results for the years 2030, 2040, 2050, and 2060 for the immigrant population of Norway and their Norwegian-born children broken down by age and sex. We distinguish immigrants and their children grouped by three categories representing country background: 1. West European countries plus the United States, Canada, Australia, and New Zealand; 2. EU-member countries in Central and Eastern Europe; 3. other countries. The remaining population forms a seventh population subgroup.

Important conclusions from the deterministic projections by Statistics Norway were a strong increase in the size of the immigrant population (more specifically those who belong to group 3) and of Norwegian-born children of immigrants. Another conclusion is that the immigrant population will age quite strongly (the native population has shown increasing shares of elderly for many decades already). Our prediction intervals to 2060 are narrow enough to rely on these conclusions. However, uncertainty in predictions for the age structures of immigrants and their children is so large that one should be very cautious when using prediction results that include age detail for one-year age groups. Aggregation into larger age groups is recommended, although uncertainty remains considerable. For the population as a whole (irrespective of migrant background), forecasts for the age structure in one-year age groups are reliable up to around 2040, except for children born after 2022. For later years, the intervals become very wide for all ages.

Meanwhile, one should keep in mind that these results are based upon two important assumptions: our best guess is the trajectory predicted by Statistics Norway, and the variation in future numbers is similar to the variation as observed in the past twenty years.

### Supplementary Information

Below is the link to the electronic supplementary material.

Supplementary file1 (PDF 488 kb)

## Footnotes

1 Croatia switched from country group 3 to country group 2 upon gaining EU membership in 2013.

2 Regional projections for the population in municipalities were published in Leknes and Løkken (2022).

3 There are four additional scenarios, primarily used for analytical purposes. Key assumptions here are constant immigration, constant life expectancy, no international migration, and equal immigration and emigration.

4 This is equivalent to Ordinary Least Squares (OLS) estimation. Construct a design matrix consisting of vectors of dummy variables, one for each age and one for each year. The estimates $\hat{\beta }_{x,s}^{OLS}$ of the age effects are identical, up to an additive constant, to the average of observed β-values across time, i.e. $\hat{\beta }_{x,s}^{OLS}$ equals a constant plus $\sum\nolimits_{t} {\beta (x,\;s,\;t)/22}$. The constant depends upon choice of reference year, and whether or not the model includes an intercept. Similarly, estimated time effects are identical, up to a constant, to the average of observed β-values across ages.

5 The Total Fertility Rate of African women living in Norway was 2.78 in 2017, 2.55 in 2018, 2.40 in 2019, 2.20 in 2020, and 2.15 in 2021. For women from Asia (incl. Turkey) the decline was weaker (https://www.ssb.no/en/statbank/table/12481/tableViewLayout1/).

## References

[CR1] Alders M (1999). Stochastische huishoudensprognose 1998–2050 [Stochastic household forecast 1998–2050]. Maandstatistiek Van De Bevolking.

[CR2] Alders M (2001). Huishoudensprognose 2000–2050: Veronderstellingen over onzekerheidsmarges [Household forecast 2000–2050: Assumptions on uncertainty intervals]. Maandstatistiek Van De Bevolking.

[CR3] Alders M (2005). Allochtonenprognose 2004–2050: Belangrijkste uitkomsten [Projections of the foreign-origin population in the Netherlands 2004–2050: Main results]. Bevolkingstrends.

[CR4] Alho, J. (1998). A stochastic forecast of the population of Finland. Reviews 1998/4. Statistics Finland.

[CR5] Alho J, Alders M, Cruijsen H, Keilman N, Nikander T, Pham DQ (2006). New forecast: Population decline postponed in Europe. Statistical Journal of the United Nations Economic Commission for Europe.

[CR6] Alho J, Keilman N (2010). On future household structure. Journal of the Royal Statistical Society Series A.

[CR7] Alho J, Spencer B (2005). Statistical Demography and Forecasting.

[CR10] Booth H (1984). Transforming the Gompertz for fertility analysis: The development of a standard for the relational Gompertz. Population Studies.

[CR11] Brass W, Brass W (1971). On the scale of mortality. Biological Aspects of Demography.

[CR13] Christiansen SG, Keilman N (2013). Probabilistic household forecasts based on register data—The case of Denmark and Finland. Demographic Research.

[CR14] Coale A, Trussell J (1974). Model fertility schedules: Variations in the age structure of childbearing in human populations. Population Index.

[CR15] Coleman D, Scherbov S (2005). Immigration and ethnic change in low-fertility countries—Towards a new demographic transition?.

[CR9] De Beer, J., & Alders, M. (1999). Probabilistic population and household forecasts for the Netherlands. Joint Economic Commission for Europe–EUROSTAT Work Session on Demographic Projections, Perugia, 3–7 May (Working paper 45).

[CR16] De Beer J (2011). A new relational method for smoothing and projecting age specific fertility rates: TOPALS. Demographic Research.

[CR17] De Beer J (2012). Smoothing and projecting age-specific probabilities of death by TOPALS. Demographic Research.

[CR18] Foss A (2012). Stokastiske befolkningsprognoser for Norge 2012–2060 [Stochastic population forecasts for Norway 2012–2060]. Økonomiske Analyser.

[CR19] Fuchs J, Söhnlein D, Weber B, Weber E (2018). Stochastic forecasting of labor supply and population: An integrated model. Population Research and Policy Review.

[CR21] Jacobs D, Swyngedouw M, Hanquinet L, Vandezande V, Andersson R, Horta APB, Berger M, Diani M, Ferrer AG, Giugni G, Morariu M, Pilati K, Statham P (2009). The challenge of measuring immigrant origin and immigration-related ethnicity in Europe. International Migration and Integration.

[CR22] Keilman, N., Pham, D.Q., & Hetland, A. (2002). Why population forecasts should be probabilistic—illustrated by the case of Norway. *Demographic Research* 6–15 May 2002, 409 – 454.

[CR23] Keilman, N. (2020). A probabilistic forecast for the population of Norway. In: A. Syse, M. Thomas & R. Gleditsch *Norway’s 2020 population projections: National level results, methods and assumptions* (pp. 177–182). Statistics Norway.

[CR24] Keilman N (2016). A combined brass-random walk approach to probabilistic household forecasting: Denmark, Finland, and the Netherlands 2011–2041. Journal of Population Research.

[CR25] Lee R, Tuljapurkar S (1994). Stochastic population forecasts for the United States: Beyond high, medium and low. Journal of the American Statistical Association.

[CR26] Leknes, S., & Løkken, S.A. (2022). Befolkningsframskrivinger for kommunene 2022 [Population projections for municipalities 2022]. Report 2022/30. Statistics Norway.

[CR28] Makridakis S, Hyndman RJ, Petropoulos F (2019). Forecasting in social settings: The state of the art. International Journal of Forecasting.

[CR29] Mead R (1965). A generalised logit-normal distribution. Biometrics.

[CR30] Preston, S., Heuveline, P., & Guillot, M. (2001). *Demography: Measuring and modelling population processes*. Blackwell.

[CR31] Rees, P. (2009). Ethnic population projections: A review of models and findings. Paper presented at the Seminar on Multi-attribute analysis and projections of ethnic populations, Quantitative Methods in the Social Sciences, Seminar Series 2 (European Science Foundation), Jevnaker, Norway, 3–5 June 2009.

[CR32] Scherbov, S., & Ediev, D. (2007). Probabilistic household projections based on an extension of headship rates method with application to the case of Russia In: *Joint Economic Commission for Europe–EUROSTAT Work Session on Demographic Projections*, Bucharest, 10–12 October (Working paper 16).

[CR33] Statistics Norway (2022). Statbank Table nr 06913: Population and population changes, by contents and year.

[CR34] Texmon, I. (1992). Norske befolkningsframskrivinger 1969–1990 [Population projections for Norway 1969–1990]. In: O. Ljones, B. Moen, & L. Østby (eds.) *Mennesker og modeller*. Sosiale og Økonomiske Studier no. 78. Statistics Norway.

[CR35] Thomas, M.J., & Tømmerås, A.M. (2022). Norway’s 2022 national population projections: Results, methods and assumptions. Report 2022/28. Statistics Norway.

[CR36] United Nations (2022). World Population Prospects 2022: Summary of Results. UN Department of Economic and Social Affairs, Population Division DESA/POP/2022/TR/NO. 3.

[CR37] Vanella P, Heß M, Wilke CB (2020). A probabilistic projection of beneficiaries of long-term care insurance in Germany by severity of disability. Quality and Quantity.

[CR38] Vassenden, K. (2015). Om kvaliteten på den norske inn- og utvandringsstatistikken [On the quality of the Norwegian immigration and emigration statistics]. Notater 2015/17. Statistics Norway.

[CR39] Wilson, T. (2013b). The sequential propensity household projection model, *Demographic Research*, 28(24), 681–712. http://www.demographic-research.org/volumes/vol28/24/28-24.pdf.

[CR40] Wilson T (2013). Quantifying the uncertainty of regional demographic forecasts. Applied Geography.

[CR41] Wiśniowski, A., & Raymer, J. (2016). Bayesian multiregional population forecasting: England. Paper joint Eurostat/UNECE work session on demographic projections Geneva, 18–20 April 2016. https://www.unece.org/fileadmin/DAM/stats/documents/ece/ces/ge.11/2016/WP07.pdf .

[CR42] Yu, C., Ševčíková, H., Raftery, A.; & Curran, S. (2023). Probabilistic county-level population projections. *Demography*, Advance online publication 22 May 2023. 10.1215/00703370-10772782 . 10.1215/00703370-10772782 PMC11065401 37212712

[CR43] Zeng Y, Wang Z, Ma Z, Chen C (2000). A simple method for estimating α and β: An extension of brass relational gompertz fertility model. Population Research and Policy Review.

