# Supplementary material for: Can We Rely on Projections of the Immigrant Population? The Case of Norway
Source: Eur J Popul. 2023 Nov 13;39(1):33. doi: 10.1007/s10680-023-09675-2 (PMC10643711; doi:10.1007/s10680-023-09675-2)
Supplement: Supplementary file 1 — Supplementary file1 (PDF 488 kb) [file 10680_2023_9675_MOESM1_ESM.pdf]

Online appendix to “Can we rely on projections of the immigrant population? The case of Norway”

This online appendix gives supplementary information about two topics: (1) alternative model specifications and (2) simulation results for country subgroups.

1. Model specifications

We considered various modifications of model (5) in order to check the robustness of our findings.

1. We included a possible cohort effect. The data series are short, just 22 years (six years for groups I2 and C2), which makes a full cohort analysis across ages 0 – 100 (groups I, I1, and I2) or even ages 0 – 69 (groups C, C1, and C2) impossible. Instead, for groups I, I1, C, and C1 we constructed an approximate “cohort standard” $\beta^C(x)$ as the average of observed $\beta(x,t)$-values along cohort lines in the $\beta(x,t)$-table, centred on the year 2010. This means we took the average of observed $\beta(x+i,2010+i)$-values for $\beta^C(x)$, i = -10, -9, -8, …, +9, +10, +11, in other words $\beta^C(x) = \Sigma_i\, \beta(x+i,2010+i)/22$. $\beta(x,t)$-values for “missing” ages and years (an upper left triangle for ages 0 – 9 and years 1990 – 1999, and a lower right triangle for ages 101 – 116 and years 2022 - 2032) had to be ignored, and averages $\beta^C(x)$ for these ages were computed for fewer than 22 $\beta(x,t)$-values. This is the procedure for men and women of groups I and I1. Cohort standards for groups C and C1 were computed in a similar way, but involved fewer ages (0 – 69). Hence, they have missing lower right triangles for ages 70 – 80 (and years 2022 – 2032). Adding pseudo cohort standards computed along these lines does not improve the results: for each group, and both for men and women, we found that cohort standards are strongly correlated with the period standards $\beta^S(x)$ for men and women in groups I and I1 (correlations of 63 and 98 per cent). For children in groups C and C1, correlations between period and cohort standards are 91 and 99 per cent. Hence, it is not possible to asses an independent effect of the cohort standards. A possible explanation is that the effect of cohort standards is already included in model (5), because, as was noted before, this model is consistent with a model for $\beta(x,t)$ that includes an age-time interaction. The latter interaction may be viewed as a way of expressing cohort effects (e.g. Luo and Hodges 2020, and the references therein).
2. One has to be prepared for error terms d(x,t) that are auto-correlated, because d(x,t) is the difference between two error terms. One solution to this problem is to extend the model as follows

$$\Delta\beta(x,s,t) = A_1 + B_1.\beta^S(x,s) + d(x,s,t)$$

(a1)

$$d(x,s,t) = \rho.d(x,s,t-1) + u(x,s,t)\ ,$$

where u(x,t) is a random error term, and ρ is a first-order autocorrelation coefficient. We checked whether an AR(1) error term would improve the fit, and estimated model (a1) by using the Prais-Winsten estimation procedure (Greene 2003). The first-order auto-regression coefficient ρ might be different for men and women, and hence estimations were done for each sex separately. All estimates of ρ turned out to be negative, but the values were moderate to small, i.e. between -0.41 and -0.0, with an average value of -0.24. Since the time series are short (22 or 7 years) and hence a parsimonious model with few parameters is to be preferred, we decided to ignore a possible auto-regression in the error term and take the results of Table 1 as the starting point for further analysis. The consequence is that estimators for the parameters in Table 1 still are unbiased, but that standard errors and t-values are incorrect. On average (across the 24 standard errors for two sexes, two model parameters, and six groups), the standard errors in model (5) are 24 per cent higher than the standard errors of the corresponding models with auto-correlated errors. Thus, we are a bit conservative, in the sense that we use standard errors of the estimates that are a little high. This is appropriate, given the often-observed underestimation of uncertainty in the prediction of densities (Armstrong et al. 2015, Makridakis et al. 2019).

3. Some support for the decision to ignore possible auto-correlation in the error term comes also from re-estimating model (5) using the Newey-West method (Greene 2003). This approach computes robust standard errors for the estimated model parameters taking both heteroscedasticity and auto-correlation of the error term into account. Following usual practice, we specified a maximum lag of $T^{0.25} = 22^{0.25} = 2.17 = 2$ years, where T is the number of years in the data series. Estimates of A_1 and B_1 were the same as those in Table 1, as expected, whereas Newey West standard errors were approximately 10 – 20 per cent lower than the robust standard errors underlying the t-values in Table 1.
4. A second attempt to improve the model was to add a quadratic term $[\beta^S(x,s)]^2$ for the standard age profile as an independent variable to model (5). In four out of six cases (groups I, I1, I2, and C), the estimate of the coefficient of the quadratic term was not significantly different from zero, at the five per cent level. For group C1, the estimate of the linear term $[\beta^S(x,s)]$ became non-significant, while that of $[\beta^S(x,s)]$ was significant (robust Student t-value equal to 3.4). Only in the case of group C2 was there a significant contribution of both the linear and the quadratic standard age profile. Since the model improved not systematically by adding a quadratic term, we decided to use a linear term only, as in expression (5).
5. We checked if ignoring data for some early or recent years would have a strong impact on the estimates. We found very little effect for estimates of groups I, I1, C, and C1, when data for the years 2000 and 2001, or for 2020 and 2021 are omitted from our data series. For groups I2 and C2, the estimates in Table 1 are based on data for the years 2015 – 2021 only. With so few years of data, it is not useful to re-estimate the model with the years 2015 – 2016, or 2020 – 2021 omitted.
6. We have used data for the years 2000 – 2021 (2015 – 2021 for groups I2 and C2). During that period, there were several shocks in international migration flows to and from Norway. These shocks may have had an effect on immigrant shares in later years. In 2020, the COVID-19 pandemic led to strong travelling restrictions, which limited international migration movements. In 2015, the war in Syria caused many

refugees and asylum seekers in Europe. For Norway, this implied a large immigration flow from country group 3. Finally, the enlargement of the European Union in 2004 and later years led to increased labour immigration to Norway from country group 2. One may account for such a shock by including a dummy variable in the model, which takes the value one in the year the shock occurred, and zero for all other years. When the dummy variable turns out to be significant, it will reduce the residual variance of the model. Dummy variables of this kind were very useful in the models for immigration to Norway analysed by Cappelen et al. (2022). Nevertheless, we have not included them in our model (5). The main reason is that one cannot exclude the possibility that there will be events in the future that have a similar bearing on immigrant shares as those mentioned above. Hence, we do not attempt to reduce the residual variance, on purpose. Again, this is appropriate, as it reflects a cautious and conservative attitude towards density forecasting (Armstrong et al. 2015, Makridakis et al. 2019).

2. Simulation results for three country groups

The median forecast in Table A1 and the expected value/Medium Variant suggest a slight increase in numbers of immigrants from country group 1, although the growth seems to flatten out by 2050. However, we are uncertain whether there will be an increase, because the 80 per cent prediction interval to 2060 covers the current value of 167,000. The 80 per cent interval is 38.0 per cent wide, relatively speaking. In other words, uncertainty is larger for this sub-group than for all immigrants in Table 4 of the main text, as one could expect.

Table A1. Number of immigrants from country group 1 in 2022 (registered), and 2030, 2040, 2050, 2060. Median value, lower and upper bounds of 67 per cent and 80 per cent prediction intervals based on 3 000 simulations, Medium Variant and Low and High Variants of Statistics Norway's projection of 2022

	2022	2030	2040	2050	2060
	1000s				
Median	167	179	187	192	192
67% prediction interval		[166-192]	[169-208]	[168-217]	[166-220]
80% prediction interval		[163-196]	[164-215]	[162-227]	[158-231]
Medium Variant		179	188	193	193
Low-High interval		[175-187]	[178-209]	[176-230]	[168-252]

Statistics Norway notes a small increase for immigrants from country group 2 to around 2050, and a slight fall to 2060. Our median value in Table A2 shows the same trajectory, but the prediction intervals indicate that the development may have been very different, once we will know the actual numbers. Uncertainty is large, with a relative width of the 80 per cent interval in 2060 equal to 37.4 per cent.

Table A2. Number of immigrants from country group 2 in 2022 (registered), and 2030, 2040, 2050, 2060. Median value, lower and upper bounds of 67 per cent and 80 per cent prediction intervals based on 3 000 simulations, Medium Variant and Low and High Variants of Statistics Norway's projection of 2022

	2022	2030	2040	2050	2060
			1000s		
Median	203	224	240	247	243
67% prediction interval		[209-239]	[217-265]	[217-279]	[209-278]
80% prediction interval		[204-245]	[210-274]	[207-290]	[199-290]
Medium Variant		224	241	248	244
Low-High interval		[209-236]	[208-264]	[196-285]	[174-301]

Table 4 in the main text suggests that there will be more immigrants in the future. At the same time, Tables A1 and A2 indicate that it is not certain that the growth concerns immigrants who belong to country groups 1 or 2. Therefore, eventual growth will most probably come from the remaining group 3. The results in Table A3 confirm this. With lower bounds for the 80 per cent intervals in the years 2030 – 2060 that are well above the current number (449,000), the conclusion must be that chances are less than 10 per cent that this immigrant group will not increase. Although we can be quite sure that there will be an increase, we do not know how strong the growth will be. The reason is that the 80 per cent interval in 2060 is rather wide. It amounts to 27.9 per cent of the median value, which indicates a bit more uncertainty than the results for all immigrants in 2060 in Table 4.

Table A3. Number of immigrants from country group 3 in 2022 (registered), and 2030, 2040, 2050, 2060. Median value, lower and upper bounds of 67 per cent and 80 per cent prediction intervals based on 3 000 simulations, Medium Variant and Low and High Variants of Statistics Norway's projection of 2022

	2022	2030	2040	2050	2060
			1000s		
Median	449	552	635	700	741
67% prediction interval		[528-578]	[592-679]	[643-761]	[666-824]
80% prediction interval		[520-585]	[579-694]	[624-780]	[644-851]
Medium Variant		552	636	702	744
Low-High interval		[518-611]	[573-745]	[605-887]	[607-1032]

When the group of Norwegian-born children of immigrants is split up by country background, the increases for children in groups 1 and 2 to 2060 are quite reliable, although the numbers involved are small, and the 80 per cent intervals in 2060 are very wide, relatively speaking – 65.5 and 57.6 per cent of the median for groups 1 and 2, respectively; see Tables A4 and A5.

Table A4. Number of Norwegian-born children of immigrants from country group 1 in 2022 (registered), and 2030, 2040, 2050, 2060. Median value, lower and upper bounds of 67 per cent and 80 per cent prediction intervals based on 3 000 simulations, Medium Variant and Low and High Variants of Statistics Norway's projection of 2022

	2022	2030	2040	2050	2060
			1000s		
Median	17	20	24	26	29
67% prediction interval		[17-23]	[19-29]	[21-34]	[22-38]
80% prediction interval		[17-24]	[18-31]	[19-36]	[21-40]
Medium Variant		20	24	27	30
Low-High interval		[19-21]	[21-27]	[23-33]	[24-38]

Table A5. Number of Norwegian-born children of immigrants from country group 2 in 2022 (registered), and 2030, 2040, 2050, 2060. Median value, lower and upper bounds of 67 per cent and 80 per cent prediction intervals based on 3 000 simulations, Medium Variant and Low and High Variants of Statistics Norway's projection of 2022

	2022	2030	2040	2050	2060
			1000s		
Median	33	43	54	61	66
67% prediction interval		[36-51]	[44-65]	[48-75]	[53-81]
80% prediction interval		[34-53]	[41-69]	[45-80]	[49-87]
Medium Variant		43	54	62	67
Low-High interval		[40-46]	[46-61]	[48-72]	[49-83]

Table A6 presents results for children of group 3. These children constitute the majority of the Norwegian-born children of immigrants. Indeed, Table A6 indicates more than a doubling between 2022 and 2060, at least in terms of the median forecast. Relative uncertainty in 2060 is large: 47.0 per cent.

Table A6. Number of Norwegian-born children of immigrants from country group 3 in 2022 (registered), and 2030, 2040, 2050, 2060. Median value, lower and upper bounds of 67 per cent and 80 per cent prediction intervals based on 3 000 simulations, Medium Variant and Low and High Variants of Statistics Norway's projection of 2022

	2022	2030	2040	2050	2060
			1000s		
Median	156	198	249	292	334
67% prediction interval		[182-216]	[219-280]	[253-343]	[280-399]
80% prediction interval		[177-222]	[211-293]	[241-360]	[265-422]
Medium Variant		199	250	297	339
Low-High interval		[187-214]	[218-288]	[244-364]	[265-444]

References in appendix

Armstrong, J. S., Green, K. C., & Graefe, A. (2015). Golden rule of forecasting: Be conservative. *Journal of Business Research*, 68 (8), 1717-1731. http://dx.doi.org/10.1016/j.jbusres.2015.03.031

Cappelen, Å., Skjerpen, T., & Thomas, M. (2022). International migration: Assumptions and results. In M. Thomas & A. Tømmerås, *Norway's 2022 national population projections* (pp. 97 – 136). Statistics Norway.

Greene, W.H. (2003). *Econometric analysis* (5th ed.). Prentice Hall.

Luo, L., & Hodges, J.S. (2020). The age-period-cohort-interaction model for describing and investigating inter-cohort deviations and intra-cohort life-course dynamics. *Sociological Methods and Research* 51(3), 1164-1210.

Makridakis, S., Hyndman, R.J., & Petropoulos, F. (2019). Forecasting in social settings: The state of the art. *International Journal of Forecasting* 36(1), 15 – 28, https://doi.org/10.1016/j.ijforecast.2019.05.011.
